# Application of a New Device for Saccadic Training in Athletes

**DOI:** 10.3390/life15060947

**Published:** 2025-06-12

**Authors:** Angelina Ganebnaya, Aiga Svede, Alina Kucika, Jekaterina Berkova, Alona Purmale, Liga Puhova, Mariya Misri, Svetlana Semjonova, Davids Davis Gailitis, Atis Kovalovs

**Affiliations:** 1 Department of Optometry and Vision Science, Faculty of Science and Technology, University of Latvia, Jelgavas Street 1, LV-1004 Riga, Latvia; alina.kucika@lu.lv (A.K.);; 2 Department of Sports Games, RSU Latvian Academy of Sport Education, LV-1006 Riga, Latvia; davidsdavis.gailitis@rsu.lv (D.D.G.); atis.kovalovs@rsu.lv (A.K.)

**Keywords:** vision training, saccades, saccadic asymmetry, saccadic velocity, saccadic amplitude, EYE ROLL device, athletes

## Abstract

The aim of our study was to test the application of a new vision training device, the EYE ROLL, for home-based eye movement training in athletes. Sixty-seven participants were randomly divided into three groups: a control group (no training); an eye movement training group with no device; and a group using the new EYE ROLL device. The results of 51 participants were used for statistical analyses after a 4-week period. Before and after the 4-week period, participants underwent the same assessment procedures: a comprehensive vision examination and saccadic eye movement recording. Before training, for both 10° and 5° stimuli, all subjects showed statistically significantly larger and faster rightward saccades compared to leftward saccades. After four weeks, the control group showed increased horizontal saccadic asymmetry and a decrease in leftward saccade amplitude. However, both velocities showed asymmetry in both visits. There were larger changes in saccadic parameters for leftward saccades, but no clear changes in saccadic response asymmetry after training. There were no consistent differences between the training groups. The EYE ROLL is a novel device that may serve as a substitute training tool for saccadic enhancement and may improve the symmetry of horizontal saccadic movements after four weeks of home-based training.

## 1. Introduction

There are several types of eye movements that are used in everyday life: conjugate (saccades and smooth pursuit, which help to explore the two-dimensional visual field) and disconjugate (vergence, which helps to explore the three-dimensional visual field). All these eye movements are of great importance in sports activities, especially in team sports where athletes have to notice and follow not only a dynamic target (ball, hockey puck, frill), but also the location and movements of their teammates. The accuracy of eye movements tends to vary from person to person. Some studies claim that persons who regularly engage in active sports as well as professional athletes have better eye movement accuracy than “novices” or non-athletes [1]. Professional athletes also have better control of their eye movements [2] and even use special viewing strategies [3].

Various eye training complexes are developed to improve the speed and accuracy of eye movements, for example, a special training program called “Dr. Revien’s Eye Exercises for Athletes” [4], the video package Eyerobics [5], and the special program “Quiet eye” [6]. The system RightEye can be used both for training and evaluating eye movements thanks to an eye tracking system that is implemented in the device.

The efficiency of these training programs and vision training in general has been studied not only in adults but also in children [7,8]. Evaluation of Dr. Revien’s Eye Exercises for Athletes and the Eyerobics program [9] revealed no significant effect on visual function and eye movements. However, Adolphe et al. [10] compared eye movements for volleyball players before and after a six-month vision training complex. As a result, it was found that the accuracy of eye movements improved significantly after vision training. The accuracy of the volleyball shots improved by 7%; the achieved result was maintained for three years after vision training. The accuracy of basketball players’ free throws improved significantly in the first year after vision training with a similar training program [11]. Even after two years, the improvement in throw accuracy was 22% [11]. The effect of vision training on the accuracy of free throws in basketball was also demonstrated in later studies [12]. An improvement in athletic performance after a vision training complex was observed for football players [13], skeet shooters [14], and darts players [15]. Thus, a number of studies show that eye movement training significantly improves performance in both amateur and professional sports.

Eye movements can be trained as a complex or separately for each type of movement. Santamaria and Story [16] conducted a study training both horizontal saccades and smooth pursuit movements in two groups: young (17–31 years old) and elderly (60–78 years old) participants. As a result, no significant improvement in saccades was observed in any of the groups after a two-week training course. In turn, smooth pursuit movements improved in both age groups [16]. Bibi and Edelman [17] demonstrated an improvement in saccadic reaction time after 6–12 training sessions. Jóhannesson et al. [18] specified that saccadic training improved not only the latency but also the peak velocity. The authors assumed that after a training session, the generation of saccades becomes more automatic and requires less effort.

Recent research indicates that oculomotor behavior can be influenced through tailored training methods, reinforcing the concept of neuroplasticity in saccadic control. For example, Bibi and Edelman [17] showed that structured saccadic training can result in observable improvements in oculomotor performance, underscoring the adaptability of the neural systems involved. Additionally, studies suggest that the brain can activate specific neural pathways in anticipation of movement in a particular direction, indicating a predictive aspect in saccadic planning. These results offer a compelling basis for creating tools to aid in enhancing saccadic function.

The equipment used in vision training for athletes varies depending on its application form. For example, special equipment is used for in-office training under professional optometrist supervision. One of the most popular in-office devices for saccadic training is the Wayne saccadic fixator (currently the Binovi Touch saccadic fixator). However, new systems appear on the market, such as the Reflextion system, which works in a similar manner to the Binovi Touch saccadic fixator. The COVID-19 pandemic boosted the offer of home-based training for athletes. For example, RightEye and VisualEdge have an option for online training at home where a stimulus is demonstrated on a computer screen. Most of the previously described systems and programs are high-cost systems. Therefore, some simple offers (with video explanations) are available, e.g., on the website of the International Sports Vision Association.

However, there is a gap in terms of equipment that could be used in home-based training to improve eye movements and studies that demonstrate the efficiency of home-based training for athletes. The EYE ROLL device (Ltd. EyeRoll, Kandava, Latvia) was created as a lightweight, portable instrument for home-based eye movement training. The functioning principle is based on saccadic and smooth pursuit stimulation with moving laser light projection on the wall. The lights are set to turn on in varied sequences, requiring the user to perform eye movements in diverse directions and at different speeds and amplitudes. Unlike many existing systems (for example, Binovi Touch, RightEye, or VisualEdge), which frequently need special screen-based setups or in-office use, EYE ROLL enables athletes to complete exercises without the need for additional hardware, supervision, or expensive costs of home-based use. The aim of our study was to test the application of a new vision training device, the EYE ROLL, for home-based eye movement training in athletes.

## 2. Materials and Methods

### 2.1. Participants

The main inclusion criteria were as follows: (1) professional athletes that have sport training at least three times a week; (2) no general or ocular diseases; (3) no head or eye surgeries; (4) no medication. To ensure sufficient eye tracking results, athletes had additional inclusion criteria: (1) binocular non-corrected visual acuity at near 0.4 (LogMAR) or better, no amblyopia, no extraocular muscle problems, no manifest strabismus, and no diplopia.

A total of 67 participants (10 females and 57 males) were involved in the study. Table 1 demonstrates the distribution of various sport disciplines included in the study. The sport discipline of each participant was verified primarily through self-reporting data collected via a questionnaire. We cannot rule out that athletes may participate in more than one sport discipline. However, all athletes indicated their main sport discipline in which they not only trained intensively (at least three times a week) but also participated in corresponding competitions. Thus, all athletes involved in the study can be considered professional athletes in one sport discipline.

All participants were randomly (not considering their sport discipline but ensuring representation of all sport disciplines in each group) divided into three groups on the first visit (Visit 1): Group 1—control group (no training); Group 2—eye movement training with no device; Group 3—eye movement training with the new device (EYE ROLL). After four weeks (Visit 2), there was a 24% drop-out of participants; only 51 participants completed the study for at least four weeks. Therefore, we changed the study design and offered all participants from Group 1 an additional four weeks, but in either Group 2 or Group 3 for the following four weeks. To summarize, initially (for Visit 1 and Visit 2), Group 1 had 32 participants (median, IQR, range: 23, 7 years, 17–42 years), Group 2 had 13 participants (median, IQR, range: 21, 9 years, 15–46 years), and Group 3 had 6 participants (median, IQR, range: 17, 16 years, 14–41 years). At Visit 2 and for the following four weeks, four participants from Group 1 started eye movement training in Group 2, and 17 participants from Group 1 started eye movement training in Group 3. In an 8-week period, Group 2E (enlarged Group 2) had 17 participants (median, IQR: 21, 8 years, 15–46 years) and Group 3E (enlarged Group 3) had 23 participants (median, IQR: 22, 9 years, 14–42 years). For Group 2E and Group 3E, we analyzed results at pre-training and post-training visits (where training was for four weeks).

All study procedures were performed in accordance with the ethical standards of the institutional and/or national research committees. The Life and Medical Sciences Research Ethics Committee of the University of Latvia granted approval for the study (No. 2022/17), ensuring compliance with the 1964 Declaration of Helsinki and its subsequent amendments or equivalent ethical guidelines. Prior to their involvement, all participants and their legal guardian (only in the case of children’s participation) provided written informed consent.

### 2.2. Study Procedure

Across all visits, participants underwent the same assessment procedure: (1) a comprehensive vision examination performed by three qualified optometrists; (2) saccadic eye movement recordings via the EyeLink 1000 Plus. Before the first visit, all participants filled in a questionnaire capturing general information, information about vision correction, regular training activity, and associated information about sport performance (see the Supplementary Materials Section).

### 2.3. Vision Examination

The vision examination included objective refraction (dry autorefractometry, Huvitz HRK-1), assessment of uncorrected visual acuity with the Snellen decimal chart, assessment of subjective refraction, and evaluations of both distance and near binocular functions. The latter included the Worth four-dot suppression test, stereovision assessed by the Osterberg and Titmus tests, heterophoria measurement with the Maddox rod test, and both positive and negative fusional reserves measured with a prism ruler. We evaluated ocular motility, the near point of convergence using the RAF ruler (Haag-Streit UK Ltd., Harlow, Essex, UK), and saccadic and smooth pursuit with the NSUCO method. Accommodative function evaluation included positive and negative relative accommodation, binocular accommodative facility (±2.00 D flipper or Wick technique for participants aged 30 or older [19]), dynamic retinoscopy MEM method, and monocular accommodative amplitude. A thorough examination of both anterior and posterior eye structures was performed to rule out any anterior or posterior ocular disorders, with particular attention to dry eye syndrome. Based on the standards established by Scheiman and Wick [19], normal values for visual functions and accommodative and non-strabismic binocular abnormalities were determined.

### 2.4. Questionnaire

The questionnaires had to be filled out online prior to the first visit. It contained general questions about a participant, their vision, and their ocular condition, as well as their general health, previous treatments, surgeries, and medications. Participants had to describe their sport discipline, frequency of training, subjective state, stress level, and performance in sport. To track the compliance of participants, all participants were provided with a compliance table to capture their regular training activities and hours spent sleeping. Additionally, both training groups recorded the daily occurrences of their saccadic training time and duration.

### 2.5. Assessment of Saccadic Movements

The study used the high-resolution (500 Hz) infrared video-based EyeLink 1000 Plus (SR Research, Ottawa, ON, Canada) eye-tracking device. This device detects the pupil using the dark pupil algorithm and captures corneal reflections [20]. A BenQ Model XL2430B monitor with a 1920 × 1080 resolution and a 533 mm by 300 mm screen size was used to display visual stimuli. The camera was adjusted to be 60 cm from the participant’s eye level and the monitor was placed 93 cm away. To ensure consistent head placement while collecting data, participants used a forehead–chin rest provided by SR Research. No glasses or contact lenses were worn during the measurements. The major cause of some participants’ lack of saccadic movement data was eye makeup, such as mascara or eyeliner, which interfered with calibration and validation procedures, consequently compromising the accuracy of saccadic movement measurement.

**Figure 1:**
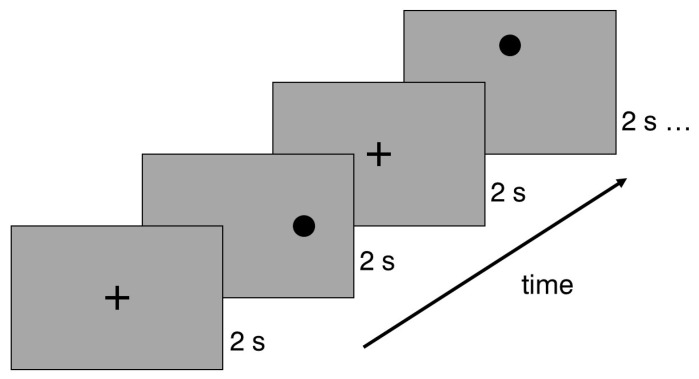
Example of a saccadic stimulus presentation sequence. The experiment started with a cross that was demonstrated for 2 s. Each saccadic stimulus was presented for two seconds, and then the fixation returned to the central cross.

The Experiment Builder software, Version 2.3.38 (SR Research, Ottawa, ON, Canada) was used to build the saccadic stimulus and its corresponding sequences. The saccadic stimulus was a black dot with a diameter of 1° (RGB 0; 0; 0), placed on a grey background (RGB 166; 166; 166) with an average luminance of 80 cd/m^2^. Each slide in the presentation sequence included a single point that was positioned either horizontally (5° or 10°) or vertically (3° or 6°) from the screen’s center. The sequence began with a central cross displayed for two seconds, followed by the two-second stimulus presentation. Subsequently, the central cross slide reappeared after each stimulus presentation (see Figure 1). The experiment was organized into three different sequences of saccadic stimuli, with each point position slide repeated three times. Thus, each sequence contained eight saccadic stimuli, arranged in a randomized order.

Prior to data collection, participants received verbal instructions to maintain a steady head position and focus on sequentially appearing dots and crosses without any head movements. The participants were also given the option to adjust their chair for optimal comfort. While blinking was permitted during the fixation phase, participants were told to avoid blinking during saccadic movement. The recordings were conducted in a well-illuminated room with an average illumination of 687 lx ± 3 lx (Illuminance Meter T-10WS).

### 2.6. Vision Training

Group 2 and Group 3 performed similar training with different types of realizations. Both saccadic and smooth pursuit stimuli were used in the training (see Table 2). In Group 2, exercises were performed standing at a distance of 1 m from the wall. For saccadic stimuli, six A3 sheets with printed tasks were given to each participant in Group 2. Participants were instructed to attach the sheets to the wall at eye level or slightly below. During the exercises, the participant had to look at the stimuli without moving their head and quickly and accurately change their fixation between targets. Smooth pursuit tasks were performed by using a red laser pointer that the participants themselves pointed at the wall. Without moving their heads, participants had to follow the flashlight with their eyes, moving the laser pointer in the described pattern.

**Figure 2:**
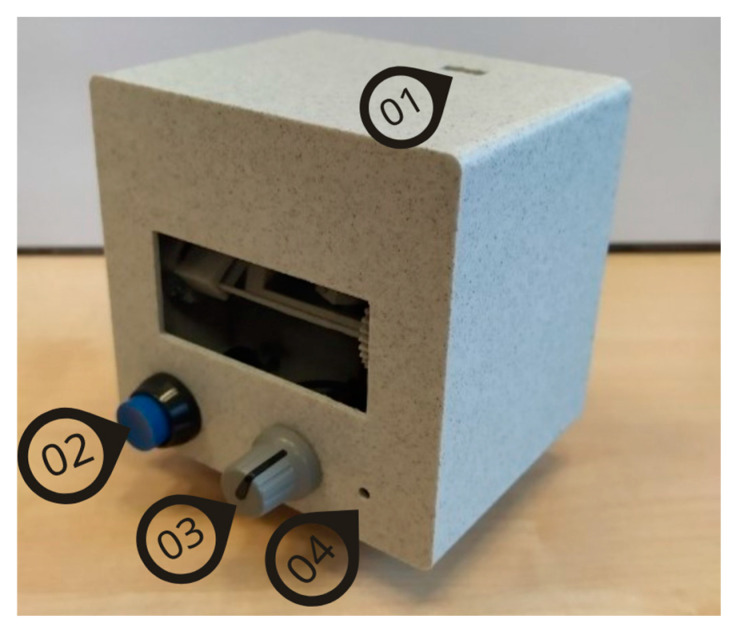
The prototype of the EYE ROLL device used in the study for Group 3 (3E): (1) USB socket; (2) control button to pause the exercise; (3) projection screen amplitude adjustment handle; (4) velocity adjustment element.

Group 3 used EYE ROLL, a novel device (see Figure 2) designed to facilitate a complex of exercises that are analogous to manual vision relaxation exercises and eye movement improvement training exercises. The participants in this study used only exercises designed for eye movement training. Most of the equipment used for similar home-based vision training is implemented on different screens (computer, smartphone, or tablet screens—RightEye, VisualEdge, and various apps), by placing different objects in the room, or by using a hand-held laser beam. The EYE ROLL device is a portable small device with the following benefits: (1) it allows eye movement exercises to be performed in a free space that is not limited by the size of the screen, so that, if necessary, the range of eye movements can be increased if the free surfaces allow it; (2) the laser beam is fully electronically controlled, so that an assistant is not required as in the case of manually controlled laser beams; (3) it is portable, i.e., easily transportable, and can be used anywhere, without being confined to specially designated training areas or systems. This equipment does not replace existing training equipment but can be considered as an additional device that could be used to improve eye movements.

All participants were instructed to ensure an ambient lighting level for better visibility of the laser dot. The dot must be projected on light-colored surfaces, such as grey or white, to ensure higher contrast. The device, when placed on a level surface using its stand, should be at least 2 m away from the projection surface, with both the user and the device maintaining this distance. The participant can take a stable standing position. The laser dot’s movement amplitude in both the horizontal and vertical directions can range from 10° to 50°. Although participants had the flexibility to adjust the amplitude based on the available projection area, the maximum amplitude was advised. By turning on the device, the training is started, and the participant has to follow with the eyes the pattern drawn with the laser dot: the dot appears in different locations for saccadic stimulation or moves with a defined speed and trajectory for smooth pursuit stimulation. By pressing the control button (see Figure 2), the participant could stop the training. When this button was pressed again, the training from the previously paused exercise was resumed.

The device employs a widened laser beam to produce a sizable point (a 1.15° red laser dot), mitigating potential risks. The EYE ROLL features a Class 3R laser (650 nm, 5 mW), which is generally considered safe for the eyes. However, extended direct or reflected exposure can pose risks, while brief exposures or radiation dispersed from non-reflective surfaces, such as walls or doors, are considered harmless. Prior to initiating device utilization, participants were advised on safety precautions: (1) avoid directly gazing into the laser beam; (2) ensure that the laser always points away from the user and other individuals and that the training surface is free from reflective objects and mirrors.

Participants in both training groups were instructed to undertake exercises at least once a day, five days a week. If participants had problems keeping their head still, it was recommended that they place a book on their head to control head movement.

### 2.7. Data Analysis

The following data were analyzed from a comprehensive vision examination: uncorrected visual acuity, subjective refraction (expressed as a spherical equivalent), phoria at distant and near fixation, fusional reserves, near point of convergence, positive and negative relative accommodation, binocular accommodative facility, and accommodative amplitude. Visual acuity was transformed from decimal units to LogMAR units for simpler analyses of visual acuity, taking mistakes (missed or incorrectly named optotypes) into account.

DataViewer (SR Research, Ottawa, ON, Canada) was used to export the saccadic movement data for each participant on each visit as Excel files. We took the binocular amplitude (in degrees), mean velocity (in degrees per second, later called velocity), duration, and peak velocity of the initial saccade from the created data file. For each stimulus location, the results of three repetitions were averaged to be used in statistical analyses. If the system recorded a blink during saccadic movement, the results of this saccadic movement were excluded from the analyses.

### 2.8. Statistical Analyses

The IBM SPSS software package, version 20.0, was used for statistical analyses. Shapiro-Wilk test was used as a normality test. The mean and standard deviation, as well as the paired *t*-test and ANOVA (with the Bonferroni post hoc test), were used if the majority of the data (50% or more) were normally distributed [21]. The median, interquartile range (IQR), and Wilcoxon signed-rank test were used for related data if most of the data (50% or more) were not normally distributed [21]. Levene’s test for equality of variance was used to evaluate the homogeneity of variances. One-way ANOVA was used to evaluate the impact of one parameter (saccadic stimulus), while the data had a homogeneous distribution of variances [21]. A mixed-model ANOVA was used to evaluate changes in saccadic parameters in relation to various affecting factors (with group as a between-subject factor, and direction, visit, sport discipline, and eye dominance as within-subject factors).

## 3. Results

### 3.1. Visual Functions Comparing Results on Visit 1 and Visit 2

The visual function of 51 participants was evaluated by comparing data before (Visit 1) and after a 4-week period (Visit 2) (see Table 3). All participants had no motility problems, good binocular single vision both at far and near distances, and stereovision at far and near distances (40–400 arc sec; median = 40 arc sec (IQR = 10)). The range of refractive error (evaluated based on the spherical equivalent of the subjective refraction) was −4.38 D to +5.25 D in the right eye and −3.63 D to +5.50 D in the left eye. The best corrected visual acuity at distant and near fixation was 0.0 (LogMAR) in all participants. Only two participants demonstrated head movements during saccades and smooth pursuits tested by the NSUCO method (2–3 points on a 5-point scale: moderate movement of the head at any time or slight movement of the head for more than 50% of the testing time) [22]. On the following visit, both improved and demonstrated either no head movements (5 points out of 5) or slight head movements (slight movement of the head less than 50% of the testing time) (4 points out of 5).

Group 1 showed statistically significant changes in phorias at distant fixation (esophoric shift) (Wilcoxon signed-rank test: Z = −2.081, *p* = 0.037), worsening of the near point of convergence (Wilcoxon signed-rank test: Z = −2.069, *p* = 0.039), an increase in the accommodative amplitude only for the right eye (Wilcoxon signed-rank test: Z = −2.516, *p* = 0.012), and a worsening of the positive fusional reserves at far distances (Wilcoxon signed-rank test: Z = −4.657, *p* < 0.001). Group 2 showed a statistically significant myopic shift in refractive error for the right eye (Wilcoxon signed-rank test: Z = −2.388, *p* = 0.017) and a decrease in positive fusional reserves at a distance (Wilcoxon signed-rank test: Z = −2.572, *p* = 0.01). There were only six participants in Group 3 on Visit 1. Therefore, we applied nonparametric methods to see the changes in visual functions. There was an increase only in the accommodative amplitude of both eyes on Visit 2 (Wilcoxon signed-rank test: right eye, Z = −2.032, *p* = 0.042; left eye, Z = −2.2.1, *p* = 0.028).

### 3.2. Visual Functions in Enlarged Training Groups

To see the effect of eye movement training on visual functions in a larger group of participants, we analyzed the results of the enlarged groups Group 2E and Group 3E (see Table 4). Group 2E (N = 17) showed a statistically significant myopic shift in refractive error for the right eye (Wilcoxon signed-rank test: Z = −2.536, *p* = 0.011) and worsening of the positive fusional reserves at far distance (Wilcoxon signed-rank test: Z = −2.549, *p* = 0.011). Group 3E (N = 23) showed an esophoric shift of phoria at near distance (Wilcoxon signed-rank test: Z = −2.644, *p* = 0.008) and an improvement in the near point of convergence (Wilcoxon signed-rank test: Z = −2.034, *p* = 0.042).

### 3.3. Saccadic Movement Difference Depending on the Sport Discipline

Fifty participants had useful saccadic data on the first visit (see Table 5). Most of the data (amplitude, mean velocity, duration, and peak velocity) were not normally distributed; therefore, the Wilcoxon signed-rank test was used for comparisons. In all participants, rightward horizontal saccades were significantly larger and faster than the leftward for both large and small amplitudes (10° amplitude: Z = −1.82, *p*(1-tailed) = 0.034; mean velocity: Z = −2.99, *p*(2-tailed) = 0.001; duration: Z = −2.046, *p*(2-tailed) = 0.04; peak velocity: Z = −4.079, *p*(2-tailed) < 0.001; 5° amplitude: Z = −3.84, *p*(2-tailed) < 0.001; mean velocity: Z = −4.38, *p*(2-tailed) < 0.001; peak velocity: Z = −4.156, *p*(2-tailed) < 0.001). Vertical saccades were symmetrical both for large and small saccades in all parameters (*p* > 0.05).

Participants were grouped by sport discipline: 15 participants in Discipline 1 (hockey, floorball, and football), 29 participants in Discipline 2 (basketball, handball, and volleyball), and six participants in Discipline 3 (shooting). More than 50% of the data (from three disciplines × four directions × two amplitudes) were normally distributed (Shapiro-Wilk *p* > 0.05); parametric tests were used for these, with homogeneity of variance being confirmed (Levene’s *p* > 0.05). No between-discipline differences were found for saccadic amplitude, mean velocity, or peak velocity when analyzing single directions (one-way ANOVA: *p* > 0.05). However, the vertical 3° downward saccadic duration differed significantly by discipline (one-way ANOVA: F(2,47) = 4.141, *p* = 0.022), with Discipline 1 showing longer durations than Discipline 3 (*p* = 0.031).

A mixed-model ANOVA (with direction as a within-subject factor and discipline as a between-subject factor) revealed that horizontal 10° rightward saccades were faster (mean velocity: F(1,47) = 4.049, *p* = 0.05; peak velocity: F(1,47) = 12.035, *p* = 0.001), with no between-discipline differences (*p* > 0.05), but the largest effects were in Discipline 2 (pairwise: *p* (mean velocity) = 0.017; *p* (peak velocity) < 0.001). For horizontal 5° saccades, rightward saccades were also larger and faster (amplitude: F(1,47) = 12.126, *p* = 0.001; mean velocity: F(1,47) = 12.231, *p* = 0.001; peak velocity: F(1,47) = 17.088, *p* < 0.001), with the largest differences in Discipline 2 (pairwise: *p* (amplitude) = 0.001; *p* (mean velocity) < 0.001; *p* (peak velocity) < 0.001).

For vertical 6° saccades, both direction and discipline had an impact (amplitude: F(2,47) = 3.779, *p* = 0.03; mean velocity: F(2,47) = 4.416, *p* = 0.017; peak velocity: F(2,47) = 6.545, *p* = 0.003). Discipline 1 showed larger and faster downward saccades (amplitude: *p* = 0.02; peak velocity: *p* = 0.003), whereas Discipline 3 had faster upward saccades (*p* = 0.014), with no differences in Discipline 2. No significant effects were found for vertical 3° saccades (*p* > 0.05). Saccadic duration showed no horizontal or vertical asymmetry in any discipline.

### 3.4. Results of Saccadic Response for Group 1 on Visit 1 and Visit 2

**Figure 3:**
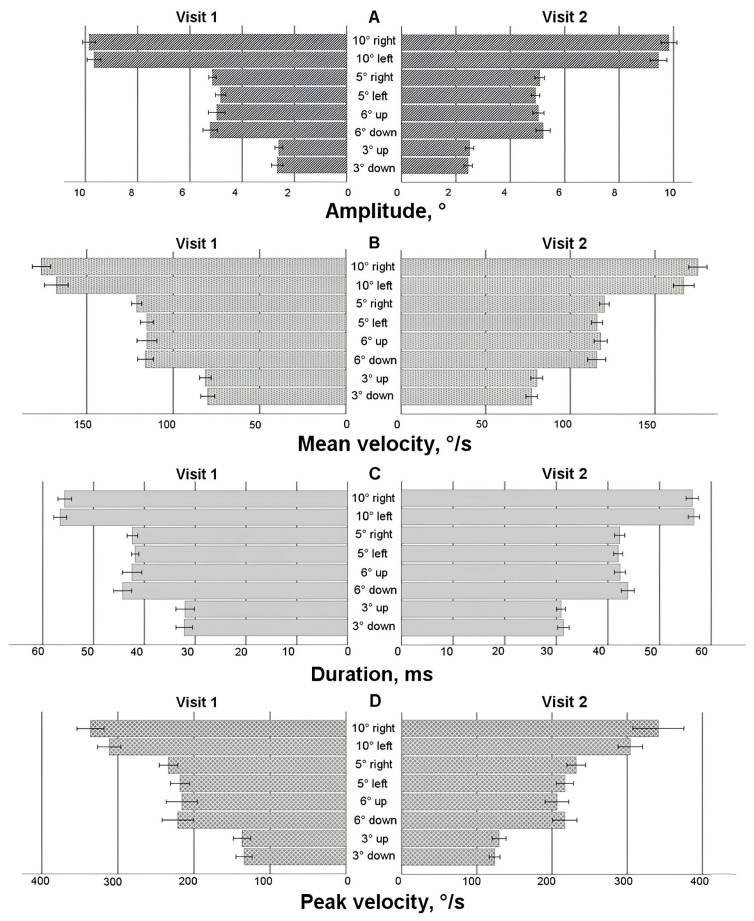
Horizontal and vertical saccade parameters for Group 1 on Visit 1 and Visit 2. (**A**) Amplitude, (**B**) mean velocity, (**C**) duration, (**D**) peak velocity.

On the second visit, 30 participants had useful saccadic data. To determine how the saccadic characteristics changed after 4 weeks in the control group (Group 1), paired *t*-tests were employed (more than 50% of the data demonstrated a normal distribution in the Shapiro-Wilk test). For horizontal saccades on the 10° left saccadic stimulus (see Figure 3A), there were shorter saccades on Visit 2 compared to Visit 1 (amplitude: *t*(29) = 2.053, *p* = 0.025 (one-sided)). For vertical 3° downward saccades, the data demonstrated shorter and faster saccadic movements on Visit 2 (amplitude: *t*(29) = 1.945, *p* = 0.031 (one-sided); peak velocity: *t*(29) = 2.096, *p* = 0.022 (one-sided)). For the duration and mean velocity, there were no statistically significant differences between Visit 1 and Visit 2.

A two-way ANOVA test with two within-subject characteristics (visit and direction) was used to check for any variations in the asymmetry of the saccadic response (a difference in saccadic parameters for one stimulus size but opposite directions). The horizontal 10° stimulus showed a difference only in the direction factor (amplitude: F(1,29) = 8.181, *p* = 0.008; mean velocity: F(1,29) = 11.250, *p* = 0.002; peak velocity: F(1,29) = 22.152, *p* < 0.001). Amplitude asymmetry was observed only on Visit 2 (*p* = 0.006); the rightward saccade was larger than the leftward (see Figure 3A). Mean velocity and peak velocity demonstrated asymmetry on both visits (*p* < 0.05); the rightward saccade was faster than the leftward.

Similar observations were for the horizontal 5° stimulus, where rightward saccadic movements were longer and faster (amplitude: F(1,29) = 8.460, *p* = 0.007; mean velocity: F(1,29) = 16.599, *p* = 0.001; peak velocity: F(1,29) = 20.637, *p* < 0.001) on both visits, except for amplitude, which demonstrated asymmetry only on Visit 1 (*p* = 0.007). No significant asymmetries in amplitude or velocity were found for the vertical stimulus. However, for the vertical 6° saccadic stimulus, a statistically significant asymmetry in duration was observed (F(1,29) = 4.320, *p* = 0.047), with this effect being more pronounced on Visit 2 (*p* = 0.041).

### 3.5. Saccadic Response in Enlarged Training Groups

**Figure 4:**
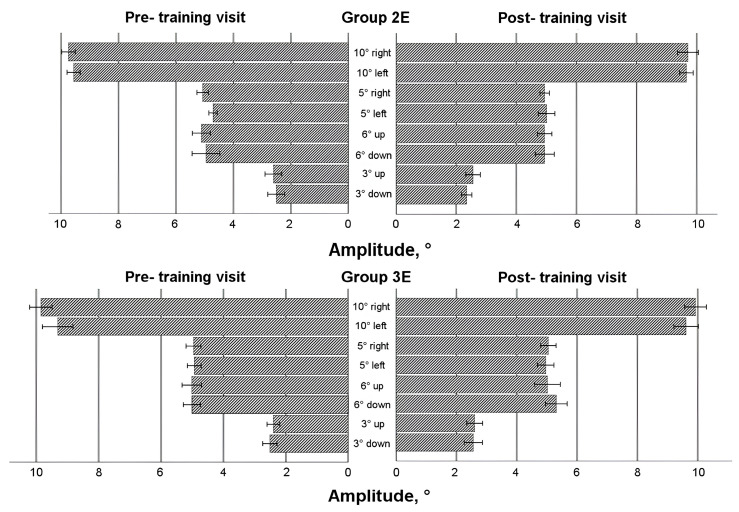
Horizontal and vertical saccade amplitude before and after eye movement training in enlarged training groups.

**Figure 5:**
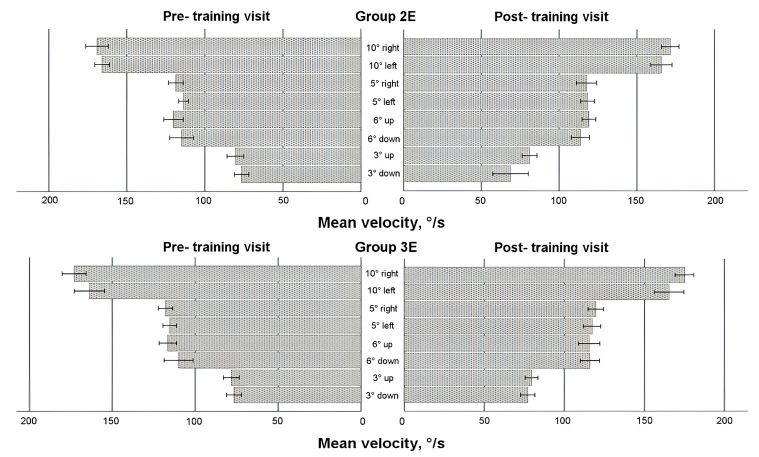
Horizontal and vertical saccadic mean velocity before and after eye movement training in enlarged training groups.

**Figure 6:**
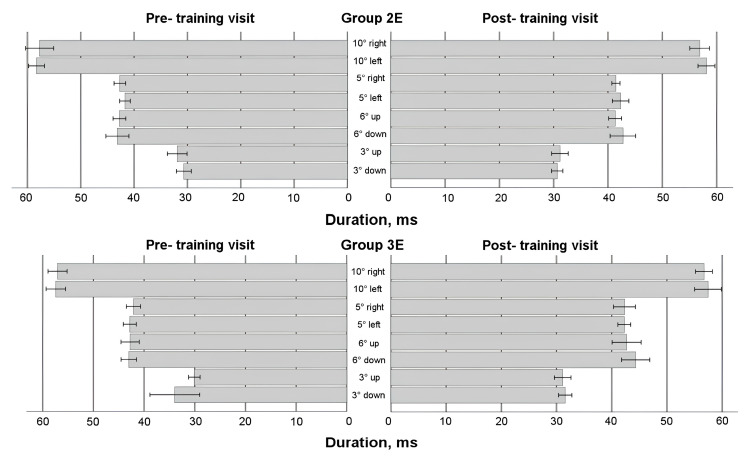
Horizontal and vertical saccadic duration before and after eye movement training in enlarged training groups.

**Figure 7:**
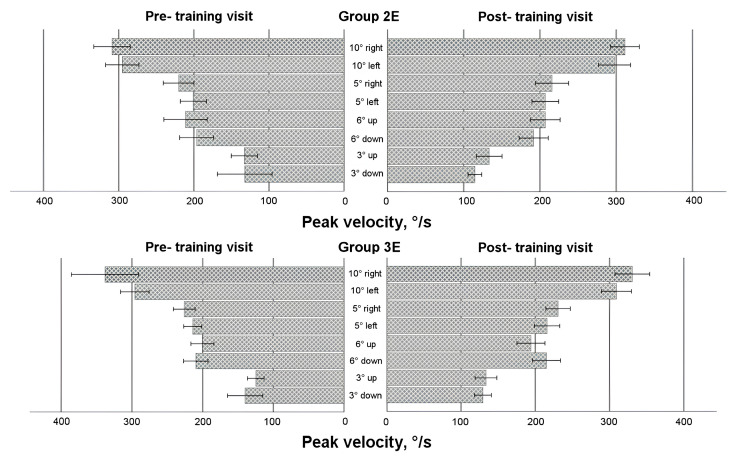
Horizontal and vertical saccadic peak velocity before and after eye movement training in enlarged training groups.

To comprehensively evaluate the effects of eye movement training, data from all participants who underwent training—regardless of whether it occurred during the first or second 4-week period—were analyzed (see Figure 4, Figure 5, Figure 6 and Figure 7). A mixed-model ANOVA (with the visit as a within-subject factor and the group as a between-subject factor) revealed that training significantly increased the leftward saccadic amplitude for both the 10° and 5° horizontal stimuli, where the saccadic amplitude was larger during the post-training visit (F(1,36) = 4.774, *p* = 0.035; and F(1,36) = 4.163, *p* = 0.049, respectively), as well as mean velocity for the 5° leftward stimulus (F(1,36) = 6.132, *p* = 0.018), where the post-training saccade was faster. No significant group-level changes were observed in saccadic duration or peak velocity after the 4-week training period (*p* > 0.05) for any horizontal direction; however, between-subject factor analyses demonstrated that participants in Group 3E exhibited significantly faster saccades (peak velocity) during the post-training visit for 10 leftward saccadic stimuli (pairwise comparison: *p* = 0.017). Similarly, individuals in Group 2E with a lower baseline peak velocity showed a larger decrease in saccadic duration for the 5° rightward horizontal stimulus (*p* = 0.041) and the 6° upward vertical stimulus (*p* = 0.037). Although no significant overall group differences were found, greater amplitude increases were observed in participants with a smaller baseline amplitude or mean velocity at pre-training: Group 3E for the 10° leftward stimulus (amplitude only) and Group 2E for the 5° stimulus (both amplitude and mean velocity). No significant changes were observed in vertical directions for the 6° and 3° stimuli (*p* > 0.05).

Two within-subject characteristics (visit and direction) were employed in the mixed model ANOVA to check for any alterations in the asymmetry of the saccadic response. Amplitude and both velocities (see Figure 4, Figure 5 and Figure 7) for the horizontal 10° stimulus showed a difference in direction factor with no group differences, with rightward saccades being larger and faster in both training groups during pre- and post- training visits (amplitude: F(1,36) = 6.122, *p* = 0.018; mean velocity: F(1,36) = 9.896, *p* = 0.003; peak velocity: F(1,36) = 12.579, *p* = 0.001). Regarding saccadic duration (see Figure 6), no significant changes were found for the 10° stimulus (*p* > 0.05).

For the horizontal 5° stimulus, all factors—visit, direction, and group—significantly influenced amplitude and duration changes (amplitude: F(1,36) = 4.436, *p* = 0.042; duration: F(1,36) = 4.665, *p* = 0.038). Notably, Group 2E showed a significant decrease in rightward saccade duration post-training, with no asymmetry detected (*p* < 0.05). However, rightward 5° saccadic amplitude was larger than leftward in Group 2E during the post-training visit (pairwise comparison: *p* = 0.007). Peak velocity for the 5° stimulus also showed a significant direction effect, with faster right-ward saccades (F(1,36) = 23.570, *p* < 0.001). Group 2E demonstrated asymmetry during the pre-training visit, and Group 3E showed asymmetry during both pre- and post-training visits, without training effects.

For the vertical 6° stimulus, no significant differences were found in amplitude, mean velocity, or duration (*p* > 0.05). However, peak velocity differed by direction and group, with downward saccades faster than upward (F(1,36) = 5.568, *p* = 0.024), and greater asymmetry observed in Group 3E during the post-training visit (pairwise comparison: *p* = 0.028). No significant differences were found for the vertical 3° stim-ulus in amplitude or duration (*p* > 0.05), but mean velocity differed by direction on both visits, with upward saccades faster than downward (F(1,36) = 6.426, *p* = 0.016). Group 2E showed post-training asymmetry with faster upward saccades (pairwise comparisons: *p* = 0.008). Peak velocity showed no significant differences overall (*p* > 0.05), though Group 2E exhibited asymmetry post-training, with upward saccades faster than downward (pairwise comparisons: *p* = 0.035).

## 4. Discussion

Our study showed larger changes in saccadic parameters for leftward saccades after saccadic training for four weeks, with no consistent difference between the training groups. Leftward saccades demonstrated increases in amplitude and velocity, as well as a decrease in saccadic duration. Such changes were not observed in the control group after a 4-week period with no training. Interestingly, the changes observed in the vertical direction were minimal and statistically insignificant. This may reflect differences in the neural mechanisms controlling eye movements in different planes. By observing changes in leftward saccades after training, we expected changes in the asymmetry of the horizontal saccadic responses. However, in the horizontal saccadic asymmetry (leftward and rightward saccadic responses), there was no unambiguous change in saccadic parameters to suggest an obvious effect of visual training. Further studies with larger numbers of participants and longer training periods are needed to better understand the neurophysiological basis of these changes and the potential of training.

The study results demonstrate that a 4-week home-based training program may considerably enhance saccadic symmetry, for both the horizontal saccadic response amplitude and the mean velocity. When participants were not engaged in any exercises (Group 1), their initial saccade’s amplitude looking to the left (for large saccadic stimuli) was reduced and became less accurate, as well as the mean and peak velocities remaining asymmetrical on both visits. The discrepancy between stimulus and saccadic amplitude was larger than the assumed accuracy (0.5°). In contrast, Group 3E improved, attaining better accuracy in the leftward saccadic amplitude. However, more precise analyses of accuracy would be useful in the following studies. We think this observation is not occasional. When participants from Group 1 were involved in eye movement training, the amplitude of their leftward saccades improved. Both enlarged training groups demonstrated improvements in horizontal saccades after exercising, with no difference between groups. Our findings are consistent with those of a previous study [18], where participants had in-office training (18 sessions) and showed improvements in saccadic amplitude and peak velocity, as well as a reduction in saccadic response asymmetry. As a consequence, even after four weeks of training, our home-based training can ensure comparable outcomes to in-office training, with the EYE ROLL device being just as effective as manual eye movement training. Therefore, we can conclude that the EYE ROLL device can ensure saccadic amplitude and velocity improvement.

The improvements observed after eye movement training can be explained not only by repeated physical practice, but also by learning how perception and movement work together. As Wulf and Lewthwaite [23] suggest, motivation and attention are key factors in the learning process. We propose that the interactive and game-like features of the EYE ROLL device may help users to stay focused and engaged during training sessions, which could enhance the overall effectiveness of the training. Although this aspect was not directly investigated in our study, the theoretical perspective may guide future research.

In sports science, stimulus salience plays an important role, as noticeable stimuli tend to capture athletes’ attention first. This influences how quickly they detect and respond to visual information using peripheral vision—an essential factor for high performance. Underwood [24] used a salience map model to show that the eyes naturally focus on visually striking areas, such as bright colors or strong contrasts. While an increase in peak saccadic velocity after training is a promising result, it is important to note that such changes are not driven solely by motor training. Previous studies [25] have shown that the perceptual and social meaning of visual stimuli can influence saccadic responses—for instance, saccades toward faces often show higher peak velocities. In sports, stimuli such as a ball or a teammate’s face naturally attract attention and can influence saccadic behavior due to their inherent salience. Future studies could benefit from using ecologically valid, sport-related stimuli to better distinguish the effects of training from attention-related responses triggered by the stimuli themselves.

We observed 24% drop-out among the participants. There were several main reasons for their dropping out. The first was non-compliance. As all training was performed on a home-based model (outside the laboratory), most of the participants reported that it was hard to fit the exercises into their daily working schedule. If participants were not performing the training as discussed in the research design, we excluded him/her from the data analysis. The second was a failure to adhere to the timetable set out in the study design. All participants were professional athletes who also regularly took part in various competitions. Consequently, some dropped out because they were unable to attend the reassessment after a four-week period. The final reason was illness: Although data collection took place after the COVID-19 pandemic (mainly in 2022) with minimum restrictions, there was still a chance of contracting the disease. Thus, some participants dropped out due to a self-quarantine period at a time when repeated measurements were planned. We cannot exclude the possibility that some of the participants who stopped their participation in the study might have experienced some complaints during the exercises, but none of the participants who continued with the exercises reported discomfort, fatigue, or challenges associated with the eye movement training (with or without the device).

Even among participants who were included in further data analysis, one of the key elements for successful home-based training was compliance. If someone trains at home, it is impossible to manage the intensity and length of the workout. The specialist can only hope the training is performed as indicated. In our study, the compliance tables helped somewhat to ensure the frequency and duration of eye movement training. According to the findings shown in these tables, both training groups’ compliance rates were high (Group 2E: 75%; Group 3E: 79%). Some participants noted that it was occasionally difficult to find time to complete the training. Some participants observed that they only remembered to complete the training just before leaving for their sports training. In such a scenario, eye movement training would be carried out less effectively and for a shorter period of time. According to compliance tables, Group 2E undertook one training per day (median; IQR = 0.2) for 10 min (IQR = 6 min). Group 3E undertook the training less often than prescribed (median 0.8 times a day; IQR = 0.4) for 13 min (median; IQR = 11 min). Previous studies demonstrated that the training session should last no less than 3 min [16]. However, better results are reached if the training session is 10 min [26], 20 min [17], or 30 min [1], with one or more short breaks for about 20 s up to 5 min between exercises [16,26]. For office-based training, the required number of sessions is 5–8 [27] to see initial gains. Eye movement improvement training should be conducted for at least 6 to 8 weeks to see a visible impact [27]. After 6–12 training sessions, for instance, a considerable improvement in saccadic latency (or response time) was noted [17]. As a result, both training groups in our study received adequate training duration and frequency. However, in our study, four weeks is the bare minimum to show early improvement but not long enough to see meaningful improvements in saccadic parameters. In order to guarantee improvements, longer training is required.

On the first visit, all participants demonstrated a larger and faster initial saccade to the right than to the left, both for small and large amplitude saccades, but no such difference was seen in the vertical direction. Even after eye movement training, the horizontal asymmetry persisted, albeit at a reduced size. According to certain research [28], asymmetry in horizontal saccades is connected to hand dominance; in the right-handed group, the saccadic latencies for the rightward saccade were considerably shorter than those for the leftward saccade, and no asymmetry was noticed in the left-handed group. We can neither confirm nor deny this statement since there were only two left-handed participants in our study. Therefore, the predominance of right-handed participants in our study might be the cause of the asymmetry in the horizontal saccadic response. The most recent research [29] shows that horizontal saccadic asymmetry could be due to naso-temporal differences in some participants and eye dominance in other participants. We can only look at eye dominance since we had no facial asymmetry measurements. Twenty-five percent of our participants had left-eye dominance (assessed by the Dolman test). No association between eye dominance and saccadic response asymmetry in the horizontal or vertical directions was found by the mixed-model ANOVA (*p* > 0.05). Additional studies are required to better explain such asymmetries in the saccadic response.

Another intriguing finding was regarding the saccadic response difference in the presented sport disciplines. Usually, there is only one sport discipline presented in the studies [7,10,11,12,13,14,15]. In Latvia, sports vision training is a young and emerging optometric field. Due to the low response rate, it was challenging to recruit volunteers for the study. This explains why there was such a wide range of sport disciplines (see Table 1). The largest group presented team sports, where eye movements, especially saccades, are crucial to viewing teammates and moving items (such as a ball or hockey puck). A small group of participants were in a shooting discipline. Discipline 2 included athletes who play basketball, volleyball, and handball. For them, horizontal saccades are the most important, and they showed larger amplitudes, and faster rightward saccades than leftward, while saccadic duration was shorter. In contrast, such asymmetry was not observed in Disciplines 1 and 3. However, athletes whose attention is downward during sports activity (Discipline 1: hockey, floorball) showed larger downward saccades (only for 6° stimuli), but shooters (Discipline 3) who must react rapidly to shooting targets (half of shooters were professional military persons) showed quicker upward saccades (only for 6° stimuli). Athletes who played basketball, volleyball, and handball showed no discernible difference in vertical saccades. Such an observation has not been made in earlier studies. We can only presume that the initial horizontal and vertical saccade has something to do with the requirements of the sports activity. To support this assertion, bigger research studies with more participants would be helpful.

There was statistically significant variation in clinical findings in each group during all study periods. Some changes cannot be considered clinically significant because they did not follow any logical clinical pattern. For example, there was myopic shift only in the right eye for participants in Group 2 (2E) but not in the left eye. There were no changes in uncorrected visual acuity. Thus, eye movement training cannot explain why there were no changes in the other eye. The only clinically logical change was in convergence and phoria in Group 3E, where the near point of convergence improved and an esophoric shift was observed for near phoria, which means exophoria decreased. This demonstrates convergence improvement at near fixation. However, the participants already had normal values of convergence on their pre-training visit. In most participants, the observed variations were in a range of corresponding measurement errors and could be considered systematic errors of the measurements or variations in repeated measurements [30,31]. For good repeatability, the measurements must be taken under the same conditions [30]. However, in our study, the time of day varied for the initial and follow-up visits (adapting to the time when the participant could arrive), as well as the visual function, the assessment of which was performed by three optometrists. Even if the techniques applied were the same, we noticed slight variability in the data even due to the optometrist factor. The range of exercises used was not initially intended to improve clinically evaluable visual functions. Based on the observed fluctuations in visual function, we cannot claim that these changes are due to the eye movement exercises.

## 5. Conclusions

To conclude, the EYE ROLL device can be successfully used as a substitute training tool for saccadic enhancements. Following four weeks of home-based training with at least one 13 min training session each day, the EYE ROLL device produced positive changes in leftward horizontal saccadic movements, making them more accurate. The results are comparable to those of manual eye movement training. It is unavoidable that the continued usage of eye movement training might have a greater effect on the saccadic response.

## Figures and Tables

**Table 1:** Number of participants and their represented sport disciplines on the first (Visit 1) and second (a 4-week period, Visit 2) visits, and in an 8-week period (extended groups).

Sport Disciplines	Visit 1 N = 67	Visit 2 N = 51	Extended Groups N = 40
Basketball	14	8	4
Volleyball	18	16	13
Handball	9	6 (4 goalkeepers)	5 (4 goalkeepers)
Floorball	14	11 (5 goalkeepers)	9 (4 goalkeepers)
Hockey	4	4 (2 goalkeepers)	4 (2 goalkeepers)
Football	2	0	0
Shooting	6	6	5

**Table 2:** Description of exercises performed by Group 2 (and Group 2E, eye movement training with no device) and Group 3 (and Group 3E, eye movement training with the new EYE ROLL device).

No	Exercise	Group 2 (2E)	Group 3 (3E)
1	Horizontal saccades	Black horizontally displaced numbered dots on two white pages (A3). Placing pages next to each other, dots made 28°, 19°, and 8° amplitude stimuli for saccades (if a participant was at a 1 m distance). The participant changed fixation sequentially and quickly from left to right and back, going through all amplitudes and naming numbers. The duration of the exercise was 1 min.	Started with the largest horizontal amplitude and decreased up to the smallest one with regular steps (largest amplitude/5). The dot was demonstrated for 500 ms, starting from the left side. Each amplitude was repeated twice. The exercise was repeated three times.
2	Vertical saccades	Black vertically displaced numbered dots on two white pages (A3). Placing pages one below each other, dots made 28°, 19°, and 8° amplitude stimuli for saccades (if a participant was at a 1 m distance). The participant changed fixation sequentially and quickly from up to down and back, going through all amplitudes and naming numbers. The duration of the exercise was 1 min.	Started with the largest vertical amplitude and decreased up to the smallest one with regular steps (largest amplitude/5). The dot was demonstrated for 500 ms, starting from the upper part. Each amplitude was repeated twice. The exercise was repeated three times.
3	Diagonal saccades	One white A3-size page with five squares (positioned in a star shape) with 4 × 4 symbols (capital letters and numbers). The distance between squares was 12–17° if a participant was at a 1 m distance. The participant changed fixation sequentially and quickly, following the star shape and reading one symbol in squares. The duration of the exercise was 1 min.	Started with the largest amplitude. The dot was demonstrated on the left side for 500 ms. Then, the dot disappeared and appeared on the right side. During the exercise, the dot followed a star shape. The exercise was repeated three times with the largest, medium, and smallest amplitudes.
4	Saccades in different directions	One white A3-size page with randomly positioned black numbers (with a distance of 2.5–11° from the center if a participant was standing at a 1 m distance) with a black cross in the center. The participant changed fixation sequentially and quickly from the center to any symbol and back to the center. The duration of the exercise was 1 min.	The dot was demonstrated in the center of the surface for 500 ms. Then, the dot appeared for 500 ms randomly at any of 16 programmed positions 25° or 12.5° from the center and reappeared in the central position afterwards.
5	Horizontal smooth pursuit in one direction		The dot appeared on the left side for 500 ms and started to move to the right with constant velocity (20°/s) making the largest amplitude. At the end, the dot stopped for 500 ms and disappeared, reappearing on the left side again. The movement was repeated five times. After a 1 s break, the movement was repeated, with the dot moving from right to left. After a 1.5 s break, the exercise was repeated with the dot moving with increased velocity: 30°/s and 40°/s.
6	Vertical smooth pursuit in one direction		The dot appeared on the upper side for 500 ms and started to move to down with constant velocity (20°/s) making the largest amplitude. At the end, the dot stopped for 500 ms and disappeared, reappearing on the upper part again. The movement was repeated five times. After a 1 s break, the movement was repeated, with the dot moving vertically up. After a 1.5 s break, the exercise was repeated with the dot moving with increased velocity: 30°/s and 40°/s.
7	Horizontal smooth pursuit in both direction	The participant held a laser pointer and drew horizontal lines on the wall. He/she started to follow the moving laser point with the eyes at the velocity that was most suitable for the eyes, gradually increasing the velocity. The duration of the exercise was 1 min.	Started with the largest horizontal amplitude and decreased up to the smallest one with regular steps (largest amplitude/5). The dot was demonstrated for 500 ms on the left side and started to move to the right with constant velocity (20°/s) making the largest amplitude. Each amplitude was repeated twice. The exercise was repeated three times, with the dot moving with increased velocity: 30°/s and 40°/s on each repetition.
8	Vertical smooth pursuit in both direction	The participant held a laser pointer and drew vertical lines on the wall. He/she started to follow the moving laser point with the eyes at the velocity that was most suitable for the eyes, gradually increasing the velocity. The duration of the exercise was 1 min.	Started with the largest vertical amplitude and decreased up to the smallest one with regular steps (largest amplitude/5). The dot was demonstrated for 500 ms on the upper side and started to move down with constant velocity (20°/s) making the largest amplitude. Each amplitude was repeated twice. The exercise was repeated three times, with the dot moving with increased velocity: 30°/s and 40°/s on each repetition.
9	Diagonal smooth pursuit	The participant held a laser pointer and drew a star on the wall. He/she started to follow the moving laser point with the eyes at the velocity that was most suitable for the eyes, gradually increasing the velocity. The duration of the exercise was 1 min.	Started with the largest amplitude. The dot was demonstrated for 500 ms on the left side and starts to move to the right with stationary velocity (20°/s), making the largest amplitude. The direction of motion changed following a star shape with a constant amplitude. The exercise was repeated three times, with the dot moving with increased velocity: 30°/s and 40°/s on each repetition.
10	Smooth pursuit in different directions	The participant held a laser pointer and drew any forms on the wall. He/she started to follow the moving laser point with the eyes at the velocity that was most suitable for the eyes, gradually increasing the velocity. The duration of the exercise was 1 min.	The dot was demonstrated in the center position for 500 ms. Then, the dot started to move in random directions and amplitudes. Duration of the exercise was 1 min. The exercise was repeated three times, with the dot moving with increased velocity: 30°/s and 40°/s on each repetition.
Recommended duration		15 min (with self-regulated short breaks changing the exercise).	15 min (with self-regulated short breaks by pressing the stop button).

**Table 3:** Visual function results of 51 participants at Visit 1 and Visit 2.

Visual Function	Group 1 (N = 32)		Group 2 (N = 13)		Group 3 (N = 6)	
	Visit 1	Visit 2	Visit 1	Visit 2	Visit 1	Visit 2
VA (OU) F (LogMAR)	−0.07 (0.49)	−0.10 (0.40)	−0.14 (0.48)	−0.13 (0.55)	−0.04 (0.33)	−0.04 (0.29)
SE (RE) (D)	−0.25 (0.97)	−0.25 (0.97)	0.07 (1.47)	−0.07 (1.57) *	0.00 (0.38)	0.13 (0.63)
SE (LE) (D)	−0.38 (1.19)	−0.38 (1.19)	−0.19 (1.26)	−0.19 (1.44)	0.00 (0.45)	0.00 (0.63)
Phoria F (Δ)	0.0 (1.9)	1.0 (2.8) *	−1.0 ± 2.6	−0.9 ± 2.6	0.0 (2.0)	1.0 (2.8)
Phoria N (Δ)	0.0 (4.0)	−1.0 (5.5)	−5.0 (9.0)	−7.0 (9.0)	0.0 (4.0)	0.0 (4.5)
NPC (cm)	4.5 (0.5)	4.5 (0.5) *	5.0 (2.3)	4.5 (1.5)	5.0 (2.5)	5.0 (1.0)
NFR F (Δ)	8 (4)	8 (4)	8 (2)	8 (4)	8 (4)	8 (3)
PFR F (Δ)	35 (0)	25 (10) ***	35 (3)	25 (17) **	35 (0)	25 (15)
NFR N (Δ)	12 ± 6	12 ± 5	13 ± 5	14 ± 7	12 (6)	12 (9)
PFR N (Δ)	30 (17)	25 (11)	33 (13)	35 (16)	16 (21)	35 (13)
NRA (D)	2.25 (0.50)	2.25 (0.44)	2.21 ± 0.40	2.23 ± 0.42	2.25 (0.75)	2.50 (0.88)
PRA (D)	−3.13 ± 1.80	−2.99 ± 1.56	−3.56 ± 1.78	−3.50 ± 1.58	−4.00 (2.38)	−2.75 (2.50)
AA (RE) (D)	9 (3)	11 (3) *	9 (7)	11 (5)	9 (4)	12 (7) *
AA (LE) (D)	11 ± 4	11 ± 3	10 (7)	14 (6)	10 (5)	13 (6) *
BAF (c/min)	6 (10)	7 (11)	2 (11)	3 (12)	0 (12)	9 (14)

Abbreviations: OU—both eyes; RE—right eye; LE—left eye; VA—uncorrected visual acuity; F—far; N—near; SE—spherical equivalent (of subjective correction); D—diopters; NPC—near point of convergence; NFR—negative fusion reserves (break); PFR—positive fusion reserves (break); NRA—negative relative accommodation; PRA—positive relative accommodation; AA—accommodative amplitude; BAF—binocular accommodative facility. For phoria, a positive sign represents esophoria, and a negative sign represents exophoria. Normally distributed data are represented as the mean and standard deviation and compared with the paired *t*-test. Non-normally distributed data are presented as the median and interquartile range and compared with the Wilcoxon signed-rank test. * *p* < 0.05, ** *p* < 0.01, *** *p* < 0.001.

**Table 4:** Visual function results of participants involved in vision training for four weeks throughout an 8-week period.

Visual Function	Group 2E (N = 17)		Group 3E (N = 23)	
	Pre-Training	Post-Training	Pre-Training	Post-Training
VA (OU) F (LogMAR)	−0.14 (0.49)	−0.14 (0.55)	−0.08 (0.50)	−0.16 (0.48)
SE (RE) (D)	0.13 (1.44)	0.13 (1.32) *	0.00 (1.00)	0.00 (1.25)
SE (LE) (D)	0.00 (1.44)	−0.13 (1.44)	0.00 (1.13)	0.00 (1.38)
Phoria F (Δ)	0.0 (3.5)	0.0 (3.0)	1.0 (2.0)	1.0 (2.0)
Phoria N (Δ)	−4.0 (8.0)	−6.0 (10.0)	−1.0 (4.0)	0.0 (4.0) **
NPC (cm)	4.5 (1.5)	4.5 (1.5)	5.0 (0.5)	4.5 (0.5) *
NFR F (Δ)	8 (2)	8 (2)	8 (2)	8 (2)
PFR F (Δ)	35 (8)	25 (16) *	30 (15)	25 (15)
NFR N (Δ)	13 ± 4	14 ± 7	14 ± 5	13 ± 6
PFR N (Δ)	30 (15)	35 (18)	25 (24)	30 (20)
NRA (D)	2.00 (0.50)	2.25 (0.50)	2.25 (0.50)	2.25 (0.25)
PRA (D)	−3.33 ± 1.90	−3.25 ± 2.03	−3.50 (2.75)	−3.25 (2.25)
AA (RE) (D)	10 (8)	11 (5)	10 ± 3	11 ± 3
AA (LE) (D)	10 (8)	13 (4)	11 ± 3	12 ± 3
DR (LE) (D)	0.75 (0.25)	0.75 (0.50)	0.75 (0.25)	0.75 (0.00)
BAF (c/min)	4 (11)	7 (10)	7 (12)	9 (13)

Abbreviations: OU—both eyes; RE—the right eye; LE—the left eye; VA—uncorrected visual acuity; F—far; N—near; SE—spherical equivalent (of subjective correction); D—diopters; NPC—near point of convergence; NFR—negative fusion reserves (break); PFR—positive fusion reserves (break); NRA—negative relative accommodation; PRA—positive relative accommodation; AA—accommodative amplitude; BAF—binocular accommodative facility. For phoria, a positive sign represents esophoria, and a negative sign represents exophoria. Normally distributed data are represented as the mean and standard deviation and compared with the paired *t*-test. Non-normally distributed data are presented as the median and interquartile range and compared with the Wilcoxon signed-rank test. * *p* < 0.05, ** *p* < 0.01.

**Table 5:** Horizontal and vertical saccade parameters on the first visit for all participants.

Discipline	Horizontal				Vertical			
	10° Right	10° Left	5° Right	5° Left	6° Up	6° Down	3° Up	3° Down
Amplitude								
Discipline 1 (N = 15)	9.6 ± 0.6	9.7 ± 0.5	5.0 ± 0.4	4.8 ± 0.3	4.9 ± 0.8	5.5 ± 0.7	2.6 ± 0.5	2.9 ± 0.6
Discipline 2 (N = 29)	9.9 ± 0.6	9.5 ± 0.8	5.1 ± 0.4	4.8 ± 0.5	5.0 ± 0.7	5.0 ± 0.8	2.5 ± 0.4	2.5 ± 0.6
Discipline 3 (N = 6)	9.9 ± 0.5	9.8 ± 0.2	5.3 ± 0.2	4.9 ± 0.3	5.5 ± 0.7	4.9 ± 0.5	2.8 ± 0.6	2.4 ± 0.3
All (N = 50)	9.8 ± 0.6	9.6 ± 0.7	5.1 ± 0.4	4.8 ± 0.4	5.0 ± 0.8	5.1 ± 0.8	2.6 ± 0.5	2.7 ± 0.6
Velocity								
Discipline 1 (N = 15)	169 ± 13	163 ± 20	116 ± 7	113 ± 7	116 ± 13	122 ± 14	79 ± 10	83 ± 9
Discipline 2 (N = 29)	175 ± 16	167 ± 15	121 ± 9	114 ± 10	114 ± 14	113 ± 12	80 ± 9	77 ± 11
Discipline 3 (N = 6)	176 ± 7	173 ± 9	124 ± 6	120 ± 4	127 ± 15	111 ± 8	83 ± 11	79 ± 7
All (N = 50)	173 ± 14	166 ± 16	120 ± 8	115 ± 9	116 ± 14	116 ± 13	80 ± 9	79 ± 10
Duration								
Discipline 1 (N =15)	57.0 ± 5.0	57.6 ± 3.4	42.8 ± 2.3	42.7 ± 2.6	42.0 ± 4.9	44.7 ± 5.7	32.8 ± 6.8	34.1 ± 4.5
Discipline 2 (N = 29)	56.5 ± 4.0	57.4 ± 4.4	42.1 ± 3.0	42.2 ± 1.9	43.2 ± 4.9	43.5 ± 4.8	30.9 ± 2.6	31.4 ± 4.0
Discipline 3 (N = 6)	56.2 ± 2.6	56.8 ± 3.0	42.3 ± 1.4	40.7 ± 1.9	43.7 ± 1.9	44.4 ± 3.4	32.7 ± 3.6	29.0 ± 1.4
All (N = 50)	56.6 ± 4.1	57.4 ± 3.9	42.3 ± 2.7	42.2 ± 2.2	42.9 ± 4.6	44.0 ± 4.9	31.7 ± 4.4	31.9 ± 4.2
Peak velocity								
Discipline 1 (N =15)	313 ± 52	299 ± 34	220 ± 36	206 ± 31	198 ± 48	248 ± 96	134 ± 40	140 ± 29
Discipline 2 (N = 29)	332 ± 47	308 ± 41	231 ± 33	216 ± 32	215 ± 52	214 ± 41	131 ± 23	140 ± 54
Discipline 3 (N = 6)	330 ± 37	315 ± 36	239 ± 24	223 ± 34	231 ± 51	182 ± 32	144 ± 37	112 ± 13
All (N = 50)	326 ± 47	306 ± 38	229 ± 33	214 ± 31	212 ± 50	220 ± 65	133 ± 31	136 ± 45

Data are represented as the mean and standard deviation.

## Data Availability

Data are available on request to the corresponding author.

## Supplementary Materials

The following supporting information can be downloaded at: https://www.mdpi.com/article/10.3390/life15060947/s1, A questionnaire that was filled by all participants before the first visit.
