# Supplementary material for: Application of a New Device for Saccadic Training in Athletes
Source: Life (Basel). 2025 Jun 12;15(6):947. doi: 10.3390/life15060947 (PMC12194661; doi:10.3390/life15060947)
Supplement: Supplementary file 1 [file life-15-00947-s001.zip › life-3605229-supplementary.pdf]

Questionnaire – Baseline Visit (modified from Google Forms)

This questionnaire is a part of the project "Research and Application Methodology Development of a New Preventive Eye Muscle Training and Strengthening Device EYE ROLL", which aims to create a simple device that would help to improve eye movements. Since you have passed the research criteria, fill out this questionnaire (it will take you 8-10 minutes). Do not hesitate to contact us in case of any questions: e-mail: …

1. Participant code
2. Mark your gender? (Mark only one)
   - ◯ Female
   - ◯ Male
   - ◯ Does not want to specify.
3. Age (in years)?
4. Which is your dominant hand? (Mark only one)
   - ◯ Right
   - ◯ Left
5. What is your daily occupation/profession?
6. Do you use optical correction on a daily basis (glasses, contact lenses, even if the correction is used irregularly)? (Mark only one)
   - ◯ Yes, I use both glasses and contact lenses *(Skip to Question 7)*
   - ◯ Yes, I use only glasses *(Skip to Question 9)*
   - ◯ Yes, I use only contact lenses *(Skip to Question 10)*
   - ◯ No, I do not use any optical correction *(Skip to Question 12)*
   - ◯ I do not use optical correction, but I use glasses with blue light blocking filters *(Skip to Question 12)*
7. What type of glasses do you use? (Mark only one)
   - ◯ Monofocal (one power glasses with minus or plus power including astigmatism correction)
   - ◯ Bifocal (with visible segment in the lower part of the lens)
   - ◯ Multifocal (with distance at the top and reading zone at the bottom) or special design lenses (e.g., anti-fatigue lenses)
8. What type of contact lenses do you use? (Mark only one)
   - ◯ Monofocal (one power glasses with minus or plus power including astigmatism correction)
   - ◯ Multifocal or bifocal (combining distant and near vision zones)

(Skip to Question 11)

9. What type of glasses do you use? (Mark only one)
   - ◯ Monofocal (one power glasses with minus or plus power including

astigmatism correction)

◯ Bifocal (with visible segment in the lower part of the lens)

◯ Multifocal (with distance at the top and reading zone at the bottom) or special design lenses (e.g., anti-fatigue lenses)

(Skip to Question 11)

10. What type of contact lenses do you use? (Mark only one)

◯ Monofocal (one power glasses with minus or plus power including astigmatism correction)

◯ Multifocal or bifocal (combining distant and near vision zones)

11. How do you use your optical correction? (Mark only one)

◯ All the time – regularly

◯ Only for distant vision

◯ Only for near vision

◯ Occasionally (e.g., only for driving or working at a computer)

12. Do you have (or have had) any general disease or health problem? (Mark only one)

◯ No

◯ Yes (specify)……………….

13. Do you have (or have had) any ocular disease? (Check all that apply)

◯ No

◯ Cataract

◯ Glaucoma

◯ Strabismus

◯ Other (specify)……………….

14. Did you have any eye surgery? (Check all that apply)

◯ No

◯ Cataract surgery

◯ Glaucoma surgery

◯ Strabismus surgery

◯ Refractive surgery (e.g., LASIK)

◯ Other (specify)……………….

15. Do you use any medication?

◯ No

◯ Yes (specify) …………………

16. Why would you like to improve eye movements? (Mark only one)

◯ To improve sports performance *(Skip to Question 17)*

◯ To improve shooting quality *(Skip to Question 21)*

17. What is your sports level? (Mark only one)

◯ Professional

◯ Amateur

18. What is your sport discipline?

19. How long is your experience in sport? (Mark only one)

◯ More than 10 years

◯ 5-10 years

◯ 2-5 years

◯ Less than 2 years

20. How many hours per week do you have sports training? (Mark only one)

◯ More than 10 hours per week

◯ 5-10 hours per week

◯ 2-5 hours per week

◯ Less than 2 hours per week

(Skip to Question 24)

21. What is your shooting discipline? (Mark only one)

◯ Military shooting

◯ Spots shooting

◯ Hunting

◯ Other….

22. How long is your experience in shooting? (Mark only one)

◯ More than 10 years

◯ 5-10 years

◯ 2-5 years

◯ Less than 2 years

23. How many hours per week do you have shooting training? (Mark only one)

◯ More than 10 hours per week

◯ 5-10 hours per week

◯ 2-5 hours per week

◯ Less than 2 hours per week

24. Have you ever used any vision training to improve eye movements? (Mark only one)

◯ Yes (Describe the exercises you used)

◯ No

◯ Not sure

Thank you for taking the time to fill out this questionnaire!
